# Absolute Protein Amounts and Relative Abundance of Volume-regulated Anion Channel (VRAC) LRRC8 Subunits in Cells and Tissues Revealed by Quantitative Immunoblotting

**DOI:** 10.3390/ijms20235879

**Published:** 2019-11-23

**Authors:** Sumaira Pervaiz, Anja Kopp, Lisa von Kleist, Tobias Stauber

**Affiliations:** 1 Institute of Chemistry and Biochemistry, Freie Universität Berlin, 14195 Berlin, Germany; 2 Department of Human Medicine, MSH Medical School Hamburg, 20457 Hamburg, Germany

**Keywords:** volume-regulated anion channel VRAC, volume-sensitive organic outward rectifying channel VSOR, leucine-rich repeat-containing, anion channel, subunit stoichiometry, heteromeric complex, tissue-specific protein expression, recombinant protein

## Abstract

The volume-regulated anion channel (VRAC) plays an important role in osmotic cell volume regulation. In addition, it is involved in various physiological processes such as insulin secretion, glia-neuron communication and purinergic signaling. VRAC is formed by hetero-hexamers of members of the LRRC8 protein family, which consists of five members, LRRC8A-E. LRRC8A is an essential subunit for physiological functionality of VRAC. Its obligate heteromerization with at least one of its paralogues, LRRC8B-E, determines the biophysical properties of VRAC. Moreover, the subunit composition is of physiological relevance as it largely influences the activation mechanism and especially the substrate selectivity. However, the endogenous tissue-specific subunit composition of VRAC is unknown. We have now developed and applied a quantitative immunoblot study of the five VRAC LRRC8 subunits in various mouse cell lines and tissues, using recombinant protein for signal calibration. We found tissue-specific expression patterns of the subunits, and generally relative low expression of the essential LRRC8A subunit. Immunoprecipitation of LRRC8A also co-precipitates an excess of the other subunits, suggesting that non-LRRC8A subunits present the majority in hetero-hexamers. With this, we can estimate that in the tested cell lines, the number of VRAC channels per cell is in the order of 10,000, which is in agreement with earlier calculations from the comparison of single-channel and whole-cell currents.

## 1. Introduction

Cells exploit various channels and transporters for inorganic ions and organic osmolytes to osmotically adjust their volume according to their physiological needs [1,2]. The volume-regulated anion channel (VRAC) is critically involved in regulatory volume decrease (RVD) upon hypotonicity-induced swelling of vertebrate cells and during various physiological processes [3]. VRAC is activated by cell swelling or other, isovolumic triggers through an unknown activation mechanism and conducts Cl^-^ and numerous organic solutes [2,3,4,5]. Their efflux is followed by water, leading to cell shrinkage. VRAC-mediated volume decrease plays also a role during early apoptosis [6,7], debatably during cell migration [8,9], and possibly during spermatogenesis [10]. In addition, membrane depolarization by VRAC-mediated Cl^-^ efflux upon glucose-induced cell swelling contributes to insulin secretion in pancreatic β-cells [11,12,13] and VRAC is involved in membrane potential alterations during myogenic differentiation [14]. Furthermore, VRAC participates in intercellular communication by conducting signaling molecules. Its adenosine triphosphate (ATP) permeability links it to purinergic signaling [15,16]. The conductance of excitatory amino acids such as glutamate is involved in astrocyte-neuron communication and excitotoxicity under ischemic conditions [17,18]. Besides physiological cargo, VRAC was also shown to at least partly mediate the cellular uptake of anti-cancer drugs such as cisplatin and of antibiotics such as blasticidin S [7,19].

After a long quest for the molecular identity of VRAC [20,21], the channel was found to be formed by heteromers of leucine-rich repeat-containing family 8 (LRRC8) proteins [22], with LRRC8A being the only essential subunit [22,23]. When expressed alone, LRRC8A can form anion channels [24,25] that are potentially regulated by the same cellular signaling [26], but it must heteromerize with at least one of its paralogues, LRRC8B-E, for physiological functionality [22,24,25,27]. The recently resolved structures of LRRC8 complexes confirmed the proposed hexameric arrangement of LRRC8 subunits within VRAC [28,29,30,31,32]. The subunit stoichiometry is variable and changes with the relative expression levels of the LRRC8 paralogues [27]. By sequential co-immunoprecipitation, it was shown that LRRC8A can heteromerize with more than one LRRC8 species within a VRAC complex [33]. Thus, a large number of VRACs with different subunit compositions is conceivable. 

While LRRC8A is the only essential VRAC subunit, the other subunit(s) within a VRAC hexamer influence some biophysical properties such as its single-channel conductance, the extent of rectification and the kinetics of depolarization-dependent inactivation [22,24,25,34]. Moreover, the subunit composition of VRAC is of great physiological importance. Firstly, it may influence the activity regulation of VRAC. VRAC consisting of LRRC8A/E was shown to be activated by the oxidation of intracellular cysteines, whereas this inhibited LRRC8A/C- and LRRC8A/D-containing VRAC [35]. Secondly, the substrate specificity of VRAC alters with the subunit composition, as revealed by heterologous expression of LRRC8 combinations and/or using gene-depleted cells lines. All combinations with LRRC8A mediate Cl^-^ currents [22,24]. The presence of LRRC8D additionally increases the permeability to organic osmolytes such as taurine, myoinositol and various amino acids with different charges, as well as to cisplatin and blasticidin [7,19,33]. Besides LRRC8A/D, LRRC8A/C and LRRC8A/E conduct aspartate and glutamate as well [33,36], and ATP is conducted by LRRC8A/E [27].

The physiological importance of VRAC is clear from the severe phenotype of mice lacking the essential LRRC8A subunit, including high embryonic lethality, as well as from several studies using gene deletion or downregulation of LRRC8A in a cell type-specific manner or in cell culture [10,12,13,14,18,37,38]. However, little is known about the specific roles of the other LRRC8 family members. Mice lacking LRRC8C gained less weight on a high-fat diet [39], which is consistent with the role of VRAC—and in particular of LRRC8C—in adipocyte differentiation [38,40]. As expected for the essential subunit, LRRC8A has been detected in all vertebrate tissues analyzed so far. While LRRC8B, -C and –D show also broad expression patterns, LRRC8E shows the most restricted tissue expression [3]. While there is information about the relative protein levels of a given LRRC8 paralogue between tissues, relative expression levels of the different subunits within a cell type are only available on mRNA level [35,36,41], despite the potentially high physiological relevance of the subunit ratio.

Here we describe a method using quantitative immunoblotting to measure the absolute protein amount and, importantly, the relative protein levels of the different VRAC LRRC8 subunits in a given cell type or organ. Recombinant expression and purification of glutathion-S-transferase (GST) fusion proteins with LRRC8 fragments containing the antigen site enabled a calibration of the immunoblot signals from murine C2C12 myoblasts, 3T3 fibroblasts and various organs. We found that LRRC8A is present at low levels, suggesting that VRAC complexes may contain only one or very few of this essential subunit. Our estimation of the number of VRACs per cell being in the magnitude of ~10,000 is in line with a previous calculations from electrophysiological studies.

## 2. Results

### 2.1. Recombinant Expression of LRRC8A-E Fragments 

**Figure 1:**
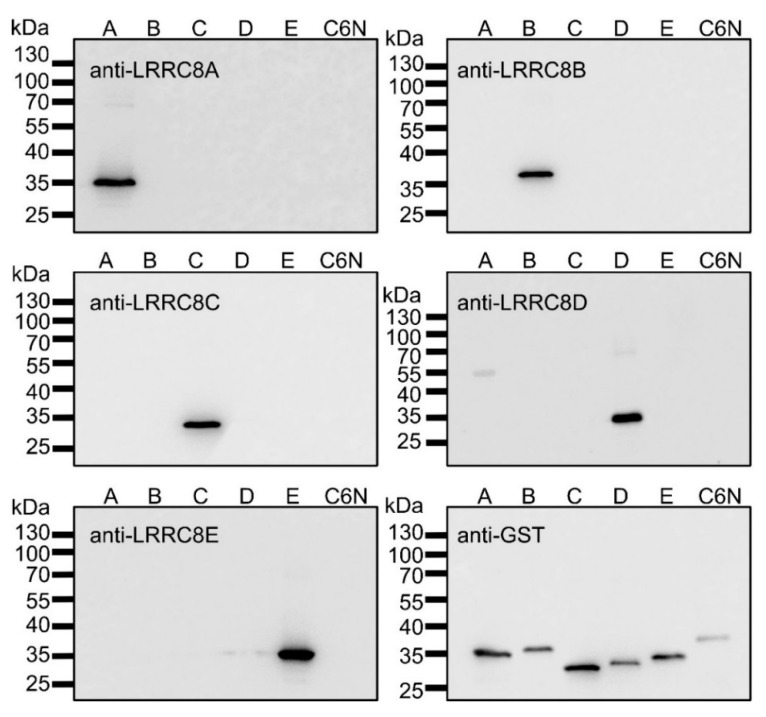
Antibody specificity against LRRC8 fragments and purity of recombinant protein. Equal amounts (10 ng/lane) of purified recombinant glutathion-S-transferase (GST) fusion proteins of LRRC8A-E fragments (A–E) and of the amino-terminal domain of ClC-6 (C6N) were probed with anti-LRRC8A-E and anti-GST antibodies. The presented blots are representative for three independent experiments.

To allow for the calibration of the immunoblot signal with the antibodies against the LRRC8 proteins, we recombinantly expressed and purified glutathion-S-transferase (GST) fusion proteins with fragments of LRRC8A-E containing the respective peptide sequence against which the antibody was generated (Table 1). The fragments of LRRC8A and LRRC8B are from an intracellular loop between transmembrane helices TM1 and TM2, and the fragments of LRRC8C-E represent the intracellularly localized carboxy-terminal ends of the proteins. Probing of all five recombinant fusion proteins of the LRRC8 fragments and of an amino-terminal domain of ClC-6 as control by immunoblot with the antibodies against LRRC8-E confirmed both the specificity of the antibodies as well as the purity of the recombinant protein (Figure 1).

### 2.2. Absolute Protein Levels of the LRRC8 Paralogues in Murine Cell Lines

**Figure 2:**
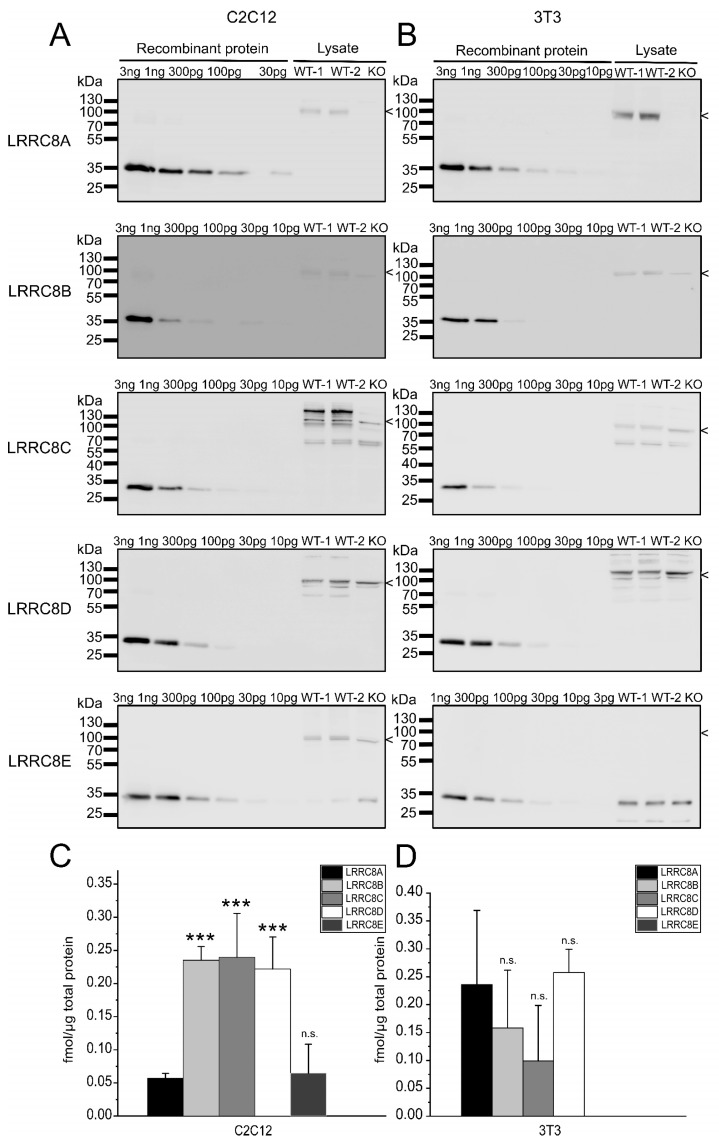
Quantification of LRRC8 protein amounts in murine cell lines. (**A**,**B**) Two replicates of whole-cell protein preparations from wild-type C2C12 (A) and 3T3 (B) cells (WT-1 and WT-2) and from a LRRC8A-deficient C2C12 and 3T3 line (KO), with 60 µg/lane, were separated by SDS-PAGE. Each blot was loaded with a dilution of recombinant GST fusion protein to calibrate for the respective antibody signal. The size of the LRRC8 proteins, as judged from the LRRC8A KO control or from comparison to data from human cells lacking all five LRRC8 proteins (Figure S1, [7]), is indicated. The blots are representative for three independent experiments. (**C**,**D**) Quantification of LRRC8A-E in C2C12 (C) and 3T3 ((D) cells from three independent blots with two lysates each. Data represent the mean from six lysates ± SD. *** *p* < 0.001, n.s. = not significant, compared with LRRC8A using one-way analysis of variance (ANOVA) with Bonferroni’s post hoc test.

To measure the protein amount of endogenous LRRC8A-E in the murine C2C12 myoblasts and 3T3 fibroblast cell lines, we separated 60 µg of whole-cell protein by SDS-PAGE and probed for the five LRRC8 proteins by immunoblot (Figure 2). Two independently prepared cell lysates for wild-type cells were tested per immunoblot. Lysate from cells deficient in the LRRC8A subunit served as control to identify the specific band for LRRC8A. Furthermore, the disruption of LRRC8A was previously shown to alter the apparent sizes of its LRRC8 paralogues [7] because LRRC8A is required for ER exit [22] and hence normal glycosylation of LRRC8B-E, as clearly observable for LRRC8C (Figure 2A,B). Probing our cell lines together with lysate of previously published HCT116 [22] and HEK293 [33] cells, either wild-type or with all five *LRRC8* genes disrupted, provided further evidence for the specificity of the selected immuno-signals (Figure S1). 

In addition to the protein from the cell lines, dilutions of the recombinant proteins ranging from 3 pg to 3 ng were loaded (Figure 2A,B). This allowed for a calibration with a linear fit in the range of the signal from the endogenous protein per blot (Figure S2; with three independent blots per protein and cell type) and hence the calculation of the absolute protein amounts for the five LRRC8 paralogues (Figure 2C,D). Interestingly, in C2C12 cells the amount of the indispensable subunit LRRC8A is approximately five-fold lower than the levels of LRRC8B, LRRC8C and LRRC8D; and similar to that of LRRC8E (Figure 2C). In 3T3 cells, LRRC8E is not expressed at detectable levels and the other subunits are present at similar numbers (Figure 2D).

**Figure 3:**
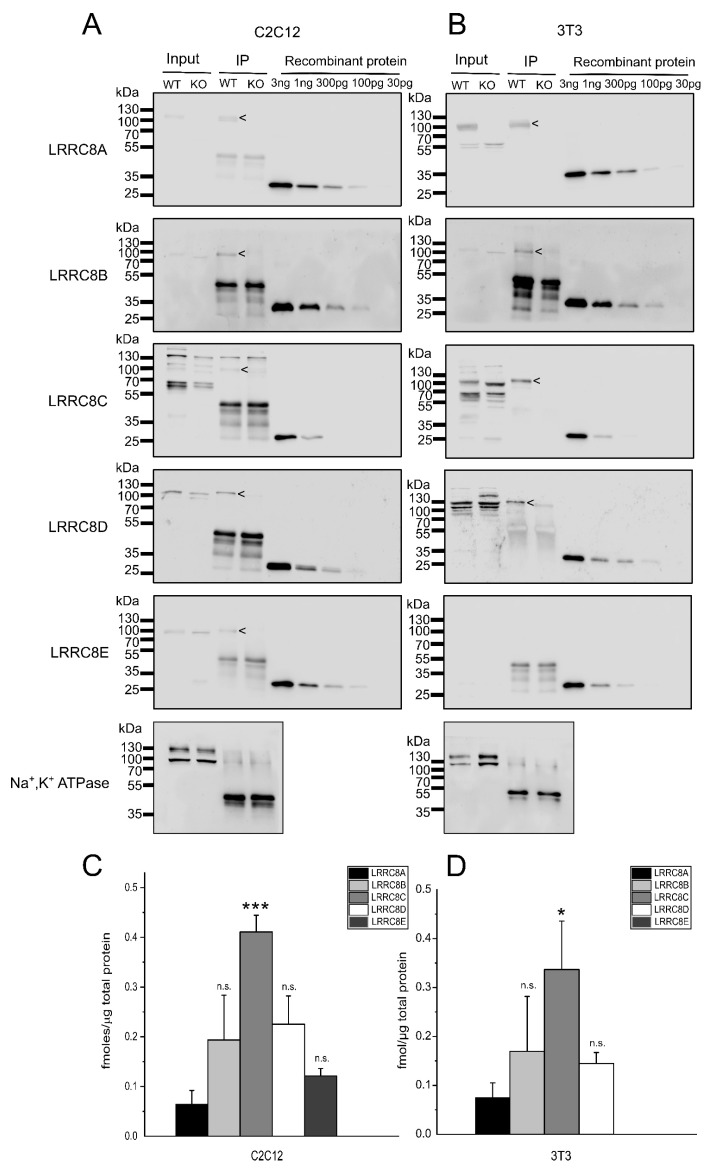
Quantification of LRRC8 protein amounts in co-immunoprecipitation with LRRC8A. (**A**,**B**) LRRC8A co-precipitated LRRC8B-E in immunoprecipitations with an LRRC8A antibody from C2C12 (A) and LRRC8B-D from 3T3 cell lysates (B), but not from the respective LRRC8A-deficient cells. The Na,K-ATPase, tested as negative control, was not co-precipitated. Lysate equivalent to 25% of input was loaded as reference (input). Each blot for LRRC8A-E was loaded with a dilution of recombinant GST fusion protein to calibrate for the respective antibody signal. (**C**,**D**) Quantification of precipitated LRRC8A-E in C2C12 (C) and 3T3 (D) cells, per µg of total protein subjected to the immunoprecipitation. Data represent mean ± SD from three independent experiments. * *p* < 0.05, *** *p* < 0.001, n.s. = not significant, compared with LRRC8A using one-way ANOVA with Bonferroni’s post hoc test.

Next, we wanted to test whether the ratios in protein levels in cell lysates reflect the subunit stoichiometries in LRRC8 complexes containing LRRC8A, which is a prerequisite for the functionality of VRAC. To this end, we immuno-precipitated LRRC8A from C2C12 and 3T3 lysates (Figure 3A,B). LRRC8B-E efficiently co-precipitated with LRRC8A, but not from LRRC8A-deficient cells. The Na,K-ATPase, tested as negative control, did not co-precipitate with LRRC8A. As for the assessment of protein amounts in the lysates of C2C12 and 3T3 cells (Figure 2), we included dilutions of the recombinant proteins to calibrate for the amounts of LRRC8A-E for each immunoblot. The relative abundance of the LRRC8 paralogues in the precipitate from C2C12 cells (Figure 3C) is very similar to that of proteins in C2C12 lysate (Figure 2C). For 3T3 cells, LRRC8A was not enriched relatively to the other subunits, even rather reduced, comparing the relative protein amounts in the precipitate (Figure 3D) with those in the cell lysate (Figure 2D). These findings are in consistence with a relatively low abundance of LRRC8A in LRRC8 hetero-hexamers.

### 2.3. Expression Analysis in Mouse Tissues 

**Figure 4:**
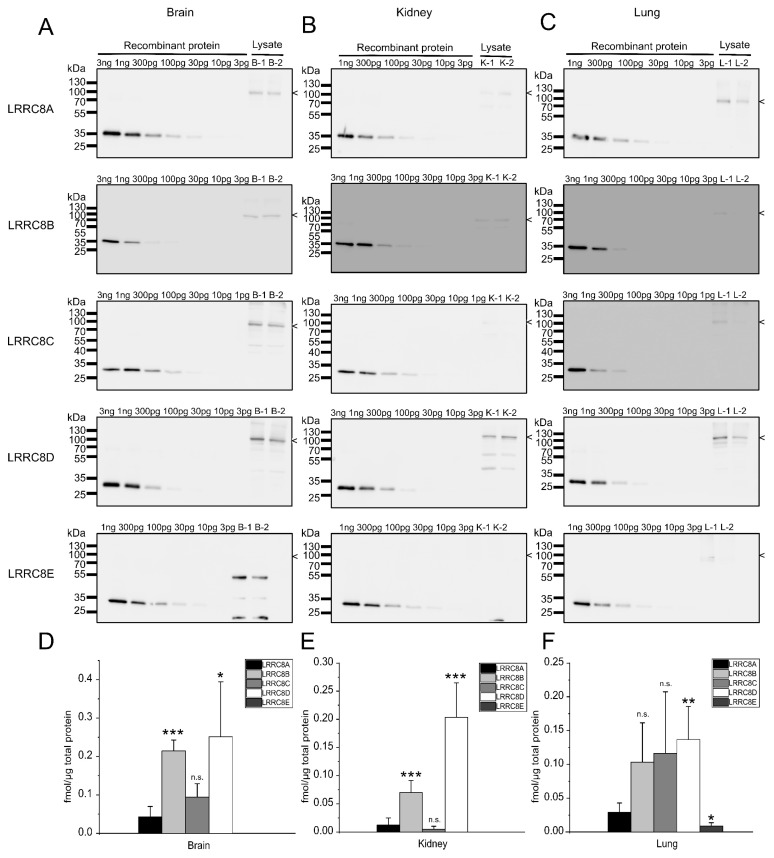
Quantification of LRRC8 protein amounts in mouse brain, kidney and lung. (**A**,**B**,**C**) Protein preparations (60 µg/lane) from brain (**A**), kidney (**B**) and lung (**C**) from two 8-weeks old animals were separated by SDS-PAGE. Each blot was loaded with a dilution of recombinant GST fusion protein to calibrate for the respective antibody signal. The size of the LRRC8 proteins, as judged from comparison to published data or data from human cells lacking all five LRRC8 proteins (Figure S1, [7]), is indicated. The blots are representative for three experiments. (**D**,**E**,**F**) Quantification of the protein amounts of LRRC8A-E in brain (**D**), kidney (**E**) and lung (**F**). Data represent mean from three independent experiments (six measurements) ± SD. * *p* < 0.05, ** *p* < 0.01, *** *p* < 0.001, n.s. = not significant, compared with LRRC8A using one-way ANOVA with Bonferroni’s post hoc test.

**Figure 5:**
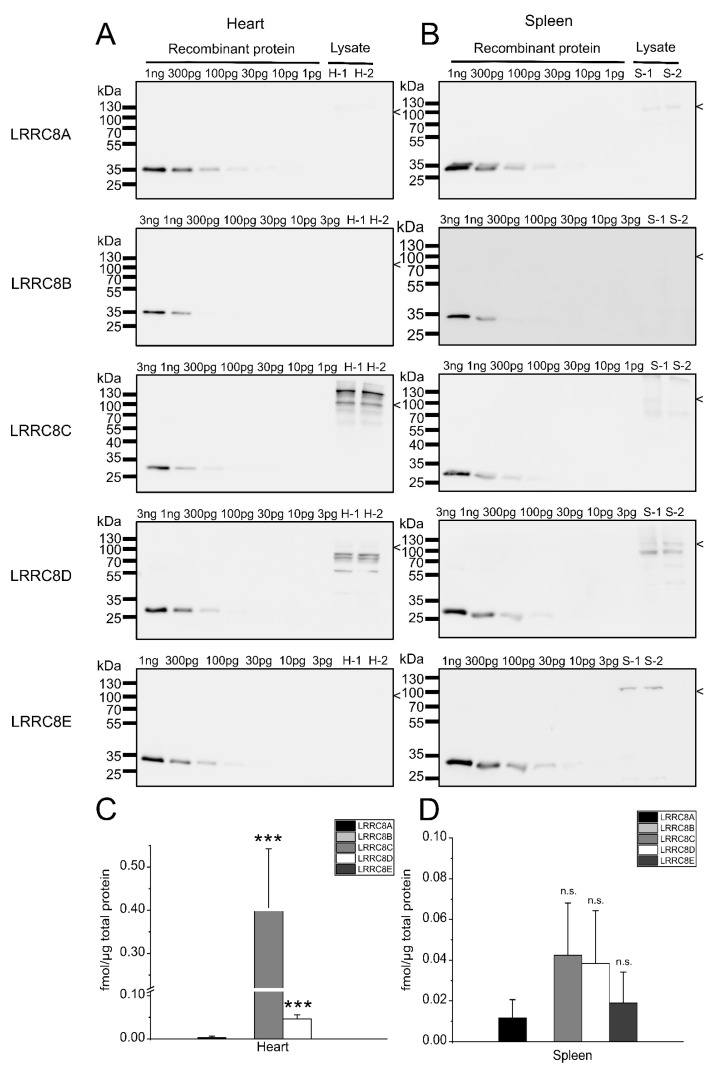
Quantification of LRRC8 protein amounts in mouse heart and spleen. (**A**,**B**) Protein preparations (60 µg/lane) from heart (A) and spleen (B) from two 8-weeks old animals were separated by SDS-PAGE. Each blot was loaded with a dilution of recombinant GST fusion protein to calibrate for the respective antibody signal. The size of the LRRC8 proteins, as judged from comparison to published data or data from human cells lacking all five LRRC8 proteins (Figure S1, [7]), is indicated. The blots are representative for three experiments. (**C**,**D**) Quantification of the protein amounts of LRRC8A-E in heart (C) and spleen (D). Data represent mean from three independent experiments (six measurements) ± SD. *** *p* < 0.001, n.s. = not significant, compared with LRRC8A using one-way ANOVA with Bonferroni’s post hoc test.

We next assessed LRRC8 protein levels in a subset of mouse tissues. To this end, we separated lysate from brain, kidney, lung (Figure 4), heart and spleen (Figure 5) containing 60 µg of protein from two 8-weeks old male mice by SDS-PAGE and included dilutions of the recombinant LRRC8 fragments to calibrate the antibody signal. 

**Figure 6:**
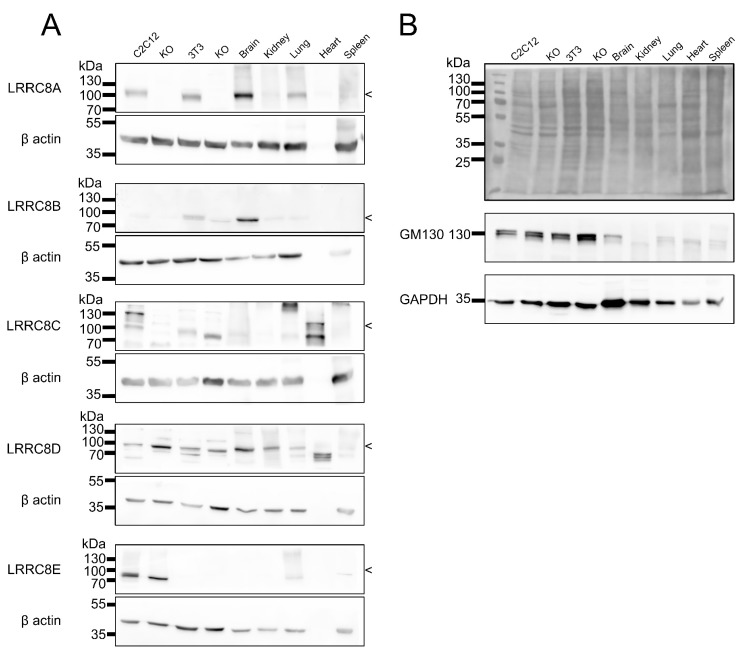
Immunoblot against the five LRRC8 proteins in lysates of different cell lines (and LRRC8A-KO of C2C12 and 3T3) and organs. (**A**) Equal amounts (60 µg protein/lane) of cell and tissue lysates were separated by SDS-PAGE, β-actin (not expressed in heart [43]) served as a loading control. The size of the LRRC8 proteins, as judged from the LRRC8A KO control or from comparison to data from human cells lacking all five LRRC8 proteins (Figure S1, [7]), is indicated. (**B**) Equal loading of wells was additionally verified by Ponceau staining after protein transfer and by probing for GM130 and GAPDH as loading controls for each immunoblot (examples shown).

As previously reported [10,13,42], the expression profiles of VRAC subunits vary between mouse tissues. As in C2C12 cells (Figure 2), in none of the tested organs is the essential LRRC8A the most abundant subunit. For example, in brain, each of LRRC8B, -C and –D are present two-fourfold (Figure 4A,D). LRRC8B shows strongest expression in brain (about 0.2 fmol/µg total protein) and about half the levels in kidney and lung (Figure 4 and Figure 6), while it was undetectable in heart in spleen (Figure 5 and Figure 6). The highest levels of LRRC8C were found in the heart (Figure 5 and Figure 6) where it is the most abundant LRRC8 paralogue. LRRC8D, is present at the same magnitude in all organs (Figure 4, Figure 5 and Figure 6). LRRC8E was only detected in lung and spleen, where it was present in similar amounts as LRRC8A (Figure 4 and Figure 5). 

Immunoblots with the cell lines and organs tested here in direct comparison largely confirmed the relative expression levels between tissues (Figure 6). 

## 3. Discussion

Here we present a method to quantify the protein levels of the five different LRRC8 VRAC subunits in cells and tissues by using recombinantly expressed and purified LRRC8 fragments to calibrate the signal in immunoblots. The LRRC8 fragments fused to GST contained longer amino acid stretches flanking the peptide sequence against which the used antibodies were raised to exclude any steric hindrance due to the GST despite the denaturation. The fragments for LRRC8C, –D and –E ended with the peptide because it lies at the extreme carboxy-terminus of the respective protein, whereas the peptides for the LRRC8A and –B antibodies localize to the first intracellular loop of the respective protein. Using the appropriate dilutions of the recombinant proteins (tested by trial and error in preliminary experiments), the protein amount in the tested lysate lied within the range of the dilution which could be linearly fitted. This method clearly has several uncertainties and the absolute values determined here must be considered with caution, but they will provide a good lead of the true protein amounts.

Our study does not only allow to compare the expression of a LRRC8 protein between tissues, but also to compare the expression of all five paralogues within one organ or cell line. This is important because the subunit composition of VRAC does not only determine various biophysical properties [5], but also the physiologically relevant substrate selectivity [7,27,33,36] and regulatory mechanisms [35]. As VRAC is formed by heteromeric hexamers and LRRC8A is essential for VRAC function, it is a striking finding that, except for 3T3 cells, in all tested tissues LRRC8A represents at most one sixth of the LRRC8 proteins—often even less. Except for 3T3 and spleen, we found at least one other subunit is present in significantly higher amounts than LRRC8A. On mRNA levels, LRRC8A was previously shown to be expressed at less than one sixth of all LRRC8 RNA molecules in Jurkat cells [35]. In rat astrocytes, LRRC8A mRNA was shown to be expressed at equally low levels [41] or at similar levels to LRRC8B-D [36]. It remained unknown whether the abundance of mRNA would indeed mirror the protein levels, and these results are in agreement with the protein levels found here. As the subunit stoichiometry depends on the relative expression levels [27], the relatively low amount of LRRC8A hints towards a very low number of LRRC8A subunits in a VRAC hexamer. The ratios between LRRC8 proteins in co-precipitation with LRRC8A, where we also found relatively little LRRC8A, corroborate this notion. A requirement of only few LRRC8A subunits per hexamer may explain the larger currents when LRRC8A was diluted in LRRC8A/C co-expression [44] and strong suppression of endogenous VRAC currents by overexpression of LRRC8A [22,23]. 

It remains to be investigated whether the non-LRRC8A subunits have preferences for certain interactions or whether they mix randomly depending on abundance. We cannot exclude the presence of LRRC8 complexes without LRRC8A, which would enable a scenario where LRRC8A—although present at relatively low levels—could be the major subunit in LRRC8 heteromers. As all of LRRC8B-E require co-expression with LRRC8A to exit the ER [22], and such complexes would likely be stuck in ER and may be subject to ER-associated degradation. It has been proposed that ER-resident LRRC8B without co-expression of LRRC8A is involved in ER calcium dynamics [45], but endogenous LRRC8B rather localizes to the plasma membrane [46].

Assuming the presence of one LRRC8A per hexameric VRAC, our quantitative immunoblotting enables an estimation of the number of VRAC complexes per cell since we know the number of cells used for the protein lysate preparation. With this, we roughly estimate the presence of ~6000 for 3T3 and ~60,000 for C2C12 cells. This is in good agreement with an earlier study that measured single-channel conductance of VRACS and estimated the expression of ~10,000 functional VRAC channels per cell in T-lymphocytes [47]. 

In summary, the method presented here enables the measurement of absolute protein levels of the five LRRC8 VRAC subunits in various cells and tissues, providing a useful tool for future studies on tissue-specific roles of VRAC and on the particular functions of the non-LRRC8A subunits. It will allow further dissection of the physiologically important subunit stoichiometry, e.g. following immunoprecipitations. An important finding of our study is the relatively low abundance of the essential LRRC8A subunit, which suggests the presence of only one or two LRRC8A per hexameric VRAC. The estimated number of VRACs per cells is in agreement with an earlier estimation from an electrophysiological study.

## 4. Materials and Methods 

### 4.1. Cloning, Expression and Purification of Recombinant GST Fusion Proteins

LRRC8 fragment were amplified by PCR with the following primers on genomic DNA purified from mouse tissue with an Invisorb kit (Invitek Molecular, Berlin, Germany) and cloned into pGEX-5X-1 with EcoRI/XhoI:

LRRC8A fw 5’-GGAATTCGAGGAGAGTGACCCCAA-3’

LRRC8A rev 5’-CCGCTCGAGTTACTTCGCCTGCTCCCCT-3’

LRRC8B fw 5’-GGAATTCCTCTCCAAGTCCAAAAC-3’

LRRC8B rev 5’-CCGCTCGAGTTACTTGGCTTGTTCGCC-3’

LRRC8C fw 5’-GGAATTCTTTGAAGTCCTCCCTCC-3’

LRRC8C rev 5’-CCGCTCGAGTTAGTCTGCTTTCAT-3’

LRRC8D fw 5’-GGAATTCCAGTGTCGGATGCT-3’

LRRC8D rev 5’-CCGCTCGAGTCAAATCCCGTTTGC-3’

LRRC8E fw 5’-GGAATTCCTCAGCCGTCTGGAGCT-3’

LRRC8E rev 5’-CTCGAGCGGTCATTCCTCCTCCAT-3’

The fusion construct of GST with the amino-terminal 80 amino acids of hClC-6 (C6N) has been reported previously [48]. 

GST fusion proteins were expressed in *E.coli* BL21 for 3 h at 37 °C after induction with 0.5 mM IPTG at an OD600 of 0.8–1.0. The bacteria were harvested by centrifugation, resuspended in lysis buffer (50 mM Tris pH 7.5, 300 mM NaCl, 5% glycerol (*v*/*v*), supplemented with complete and AEBSF (0.5 mg/mL)) and lysed by sonication. Subsequently, Triton X-100 was added to a final concentration of 1% and cell debris was removed by centrifugation (40 min, 19,500× *g*, 4 °C). For GST fusion protein purification, glutathione sepharose in binding buffer (50 mM Tris pH 7.5, 300 mM NaCl, 5% glycerol (*v*/*v*), 0.1% Triton X-100 (*v*/*v*)) was added to the cell lysates and incubated under agitation for 1 h at 4 °C. The sepharose-lysate mix was centrifuged for 5 min at 500× *g* and 4 °C to obtain the flow-through fraction. The slurry was washed five times with binding buffer supplemented with complete protease inhibitor cocktail and AEBSF (0.5 mg/mL) and the bound protein was eluted from the sepharose in elution buffer (50 mM Tris-HCl, 10 mM reduced glutathion, pH 8.0). Protein concentration was measured with the BCA-assay kit (Thermo Fisher Scientific, Darmstadt, Germany) and proteins were flash-frozen in liquid nitrogen and stored at −80 °C until further use.

### 4.2. Cell Lines and Generation of Knockout Cell Lines Using the CRISPR/Cas9 Technology

3T3 mouse fibroblast cell line (American Type Culture Collection, Manassas, VA, USA; ATCC CL-173) and C2C12 mouse skeletal muscle myoblasts (American Type Culture Collection, Manassas, VA, USA) were kindly provided by P. Knaus, Freie Universität Berlin, Germany. The generation of the C2C12-deficient cell line (clone 13) was described previously [9]. For disruption of the *Lrrc8a* gene by the CRISPR/Cas9 technology in 3T3 cells, the px459-V.2 single plasmid vector was used, as previously described for C2C12 [9]: Target sgRNAs (5‘-GCCCCGGAAGGAGTCGTTGCAGG-3′) were cloned into the px459-V.2 vector and transfected into 3T3 cells using Fugene HD transfection reagent (Promega, Mannheim, Germany). Two days after transfection, the cells were treated with 10 µg/mL puromycine for two days. Subsequently, the cells were allowed to grow in DMEM medium (PAN-Biotech, Aidenbach, Germany) supplemented with 10% fetal bovine serum (FBS), 100 units/mL penicillin and 100 µg/mL streptomycin without puromycine for three days before single clone expansion. Monoclonal cell lines were raised and tested for sequence alterations using target-site-specific PCR primers (5′-CATGTATGTCTCACTACACCTAACTTGTAG-3′ and 5′-CCAGGAAGATGAGGGTGTGCA-3′) on genomic DNA followed by Sanger-sequencing and Western blot analysis to confirm the knockout of the LRRC8A protein.

HCT116 and HEK293 cells with CRISPR/Cas9-mediated disruption of all *LRRC8* genes, whose generation was described previously [22,33], were kindly provided by T.J Jentsch, FMP and MDC Berlin, Germany. The cells were maintained in McCoy’s 5A medium (HCT116; Thermo Fisher Scientific, Darmstadt, Germany) or DMEM (HEK293) supplemented with 10% FBS at 37 °C in a humidified atmosphere with 5% CO_2_.

### 4.3. Preparation of Cell and Tissue Lysates

Cells were scraped on ice, pelleted down at 2000× *g* for 5 min and resuspended in pre-cooled RIPA buffer (150mM NaCl, 50mM Tris pH 8.0, 5mM EDTA pH 8.0, 1% NP-40, 0.5% sodium deocycholate, 0.1% SDS) containing proteinase inhibitor cocktail (Roche, Basel, Switzerland), vortexed and incubated on ice for 30 min with being vortexed every 10 min. The resuspension was finally centrifuged for 10 min at 10,000× *g* at 4 °C, the resulting supernatant was mixed with SDS sample buffer and heated to 95° for 5 min. Total protein concentration of the lysate was determined using the Pierce BCA Protein Assay kit (Thermo Fisher Scientific, Darmstadt, Germany). 

To prepare tissue lysates, 8-weeks old male C57BL/6 wild-type mice were sacrificed by cerebral dislocation. The mouse organs were homogenized with Triple-Pure High Impact Zirconium Beads with a 1.5 mm diameter in RIPA buffer using a BeadBug 6 Position Homogenizer (Benchmark Scientific, Edison, USA). To remove cell debris, the samples were centrifuged for 10 min at 1000× *g* at 4 °C (Heraeus Fresco 21, Thermo Fisher Scientific, Darmstadt, Germany). The supernatant was used as whole-organ lysate and the protein concentration was determined with the Pierce BCA Protein Assay kit (Thermo Fisher Scientific).

### 4.4. SDS-PAGE and Immunoblotting

Recombinant proteins and cell and tissue lysates were separated by 10% SDS-PAGE transferred to nitrocellulose membranes (Macherey Nagel, Düren, Germany) at 200 mA for 80 min. Membranes were subsequently blocked with 5%-skim milk-TBS-T (20mM tris pH 7.6150 mM NaCl and 0.02% Tween-20) for 1 h at room temperature and incubated with the respective primary antibodies overnight at 4 °C. The rabbit antibodies against LRRC8A-E (used at 1µg/mL), kindly provided by T.J Jentsch, FMP and MDC Berlin, Germany, were described previously [7,22]. For their generation, the epitope peptides (Table 1) were coupled through N-terminally added cysteines to keyhole limpet hemocyanin and injected into rabbits. The polyclonal antibodies were affinity-purified from sera with the respective peptides and their concentrations were determined using the Pierce BCA Protein Assay kit (Thermo Fisher Scientific, Darmstadt, Germany) [7,22]. The other used primary antibodies were: rabbit anti GST (used at 1µg/mL; Cell Signaling, Frankfurt am Main, Germany; #5475S); mouse anti Na,K-ATPase α1 (clone C464.6; 1:1000; Merck, Darmstadt, Germany; #05-369); mouse anti β-actin antibody conjugated to HRP (AC-15; 1:10,000; Abcam, Berlin, Germany; #ab49900); mouse anti GM130 (clone 35; 1:300; BD Biosciences, Heidelberg, Germany; # 610822); rabbit anti GAPDH (1:2500, Cell Signaling; #2118). After washing, membranes were incubated with horseradish peroxidase (HRP)-conjugated secondary antibody (1:5000; Jackson ImmunoResearch laboratories, Ely, UK) for 1 h at room temperature, washed with TBS-T and developed by enhanced chemiluminescence reagent (HRP juice; PJK GmbH, Kleinblittersdorf, Germany) and a ChemiSmart 5000 digital imaging system (Vilber-Lourmat, Collègien, France). Proteins were quantified using Fiji software [49]. 

### 4.5. Co-Immunoprecipitation

For co-immunoprecipitation, C2C12 or 3T3 cells were lysed in 300 μL lysis buffer (150 mM NaCl, 50 mM Tris-HCl pH 7.5, 1% NP-40, 0.5% sodium deoxycholate) containing 4 mM Pefabloc (Carl Roth, Karlsruhe, Germany) and proteinase inhibitor cocktail (Roche, Basel, Switzerland) for 10 min on ice. After pre-clearing by centrifugation at 14,000 rpm for 10 min at 4 °C, the lysate was spun at 30,000× *g* for 30 min at 4 °C. Total protein concentration was subsequently determined using the Pierce BCA Protein Assay kit (Thermo Fisher Scientific, Darmstadt, Germany). 15 μg of the LRRC8A antibody was added to the cleared lysate containing 2 mg of total protein. After rotation for 1–2 h at 4 °C, 50 μL of Protein A Dynabeads (Thermo Fisher Scientific, Darmstadt, Germany) were added and rotation continued overnight at 4 °C. After four washes with 500 μL wash buffer (as lysis buffer, but 0.1% NP-40 and 0.05% sodium deoxycholate), precipitates were eluted in 100 μL Lämmli sample buffer. Proteins were separated by SDS-PAGE and analyzed by immunoblot as indicated. Experiments were repeated three times.

### 4.6. Calculation of Protein Amounts and Statistical Analysis

For each immunoblot, a calibration curve was generated with the recombinant proteins (Figure S2). With the signal of the protein of interest in the cell or tissue lysate (quantified using Fiji software [49]) within the linear range of the calibration curve, the amount of LRRC8 protein within 60 µg of total protein was calculated by multiplying the equivalent value of GST-fusion protein (calculated with the linear regression of the calibration curve) with the ratio between the molecular weight of the LRRC8 protein (LRRC8A, 100 kDa; LRRC8B, 95 kDa; LRRC8C, 100 kDa; LRRC8D, 110 kDa; LRRC8E, 100 kDa) and the GST-fusion protein (34 kDa). With the molecular weight, the molar amount per 1 µg total protein was calculated. Two independent lysates were tested per calibration curve and all experiments were performed three times, so that the procedure yielded six values per protein and cell type/organ. To estimate the number of VRACs per cell, the total protein amount was measured in lysate from a known number of cells. 

The quantitative data are presented as mean of the six measurements ± SD. *p* values were determined by a one-way analysis of variance (ANOVA) with Bonferroni’s post hoc test. *p* values are indicated according to convention: * *p* < 0.05, ** *p* < 0.01, *** *p* < 0.001, n.s. = not significant.

## Figures and Tables

**Table 1:** LRRC8 fragments containing the peptide against which the antibodies were raised (marked in bold within the protein fragment sequence). Note that the peptides for the generation of anti-LRRC8C, -D and –E antibodies localize at the extreme carboxy-termini of the proteins.

Target	Epitope Peptide	LRRC8 Protein Fragment Fused to GST
LRRC8A	QRTKSRIEQGIVDRSE [22]	EESDPKPAFSKMNGSMDKKSSTVSEDVEATVPML**QRTKSRIEQGIVDRSE**TGVLDKKEGEQAK
LRRC8B	QSLPYPQPGLESPGIESPT [7]	LSKSKTLLSTSGGSADIDASK**QSLPYPQPGLESPGIESPT**SSVLDKKEGEQAK
LRRC8C	EDALFETLPSDVREQMKAD [7]	FEVLPPELGDCRALKRAGLVV**EDALFETLPSDVREQMKAD**
LRRC8D	LEVKEALNQDVNVPFANGI [7]	QCRMLKKSGLVVEDQLFDTLP**LEVKEALNQDVNVPFANGI**
LRRC8E	LYEGLPAEVREKMEEE [22]	TLPEELGDCKGLKKSGLLVEDT**LYEGLPAEVREKMEEE**

## Supplementary Materials

Supplementary Materials can be found at https://www.mdpi.com/1422-0067/20/23/5879/s1.
