# Supplementary material for: Absolute Protein Amounts and Relative Abundance of Volume-regulated Anion Channel (VRAC) LRRC8 Subunits in Cells and Tissues Revealed by Quantitative Immunoblotting
Source: Int J Mol Sci. 2019 Nov 23;20(23):5879. doi: 10.3390/ijms20235879 (PMC6928916; doi:10.3390/ijms20235879)
Supplement: Supplementary file 1 [file ijms-20-05879-s001.pdf]

**Supplementary information for**

# Absolute protein amounts and relative abundance of volume-regulated anion channel (VRAC) LRRC8 subunits in cells and tissues revealed by quantitative immunoblotting

Sumaira Pervaiz, Anja Kopp, Lisa von Kleist and Tobias Stauber

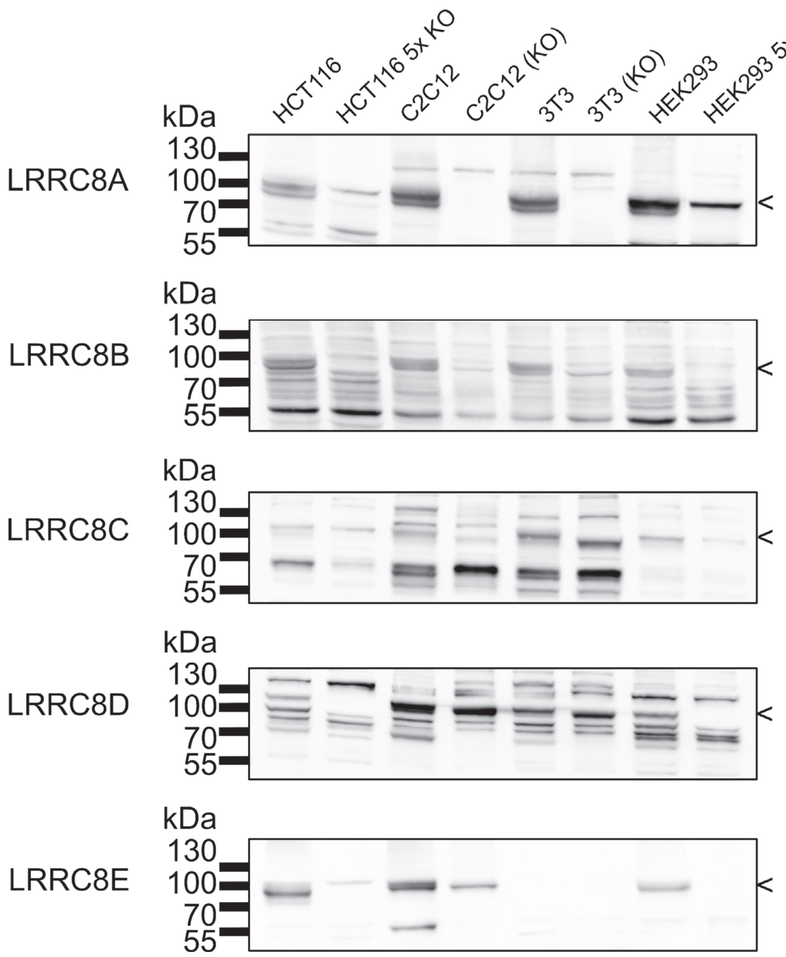


**Figure S1**. Knock-out-controlled immunoblot against the five LRRC8 proteins in lysates of different cell lines. Equal amounts (60 µg protein/lane) of cell lysates were separated by SDS-PAGE: human HCT116 and HEK293, each wild-type and quintuple knock-out of *LRRC8A-E* (5xKO), and murine C2C12 and 3T3, each wild-type and *Lrrc8a* knock-out (KO). The size of the LRRC8 proteins is indicated.

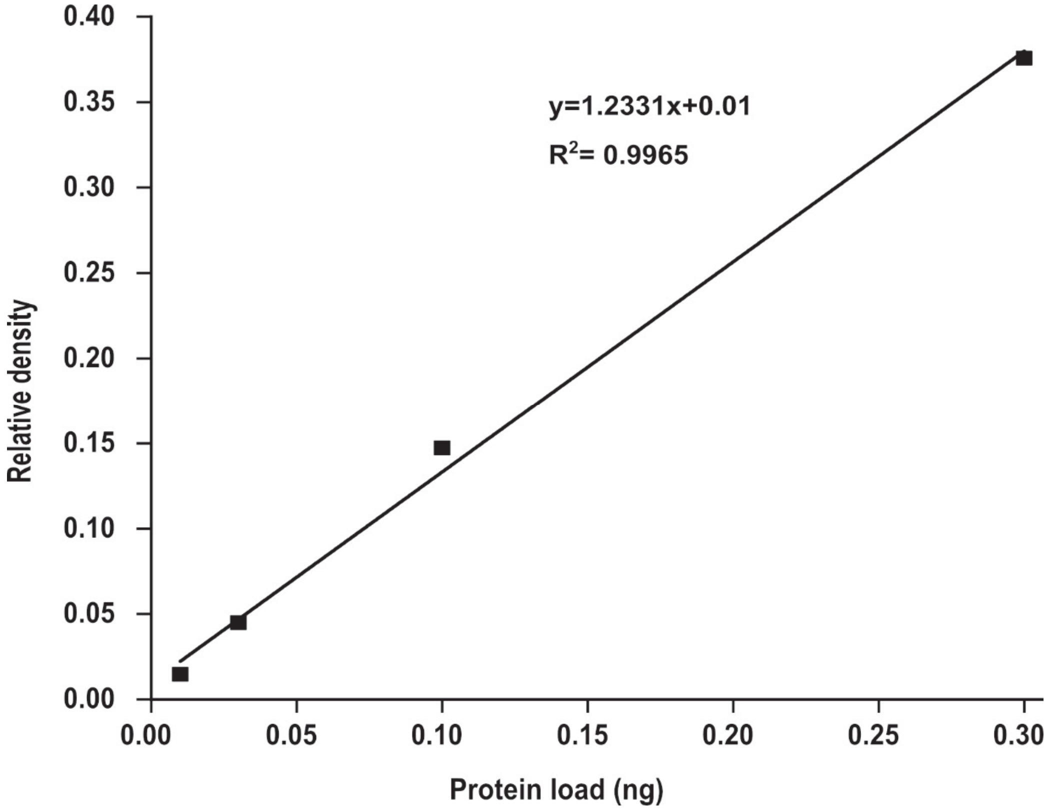


**Figure S2**. Example for the calibration of protein amounts. Shown is one out of three independent calibration curves for LRRC8A in C2C12 cells. The measured signal is plotted as a function of the amount of recombinant protein loaded. With the measured values for LRRC8A from the cell lysates lying within this linear range, the protein amount is calculated as described in the Materials and Methods section.
